# The Effect of Female Quality on Male Ejaculatory Expenditure and Reproductive Success in a Praying Mantid

**DOI:** 10.1371/journal.pone.0124209

**Published:** 2015-05-13

**Authors:** Anuradhi Jayaweera, Katherine L. Barry

**Affiliations:** Department of Biological Sciences, Macquarie University, Sydney, NSW 2109, Australia; University of Melbourne, AUSTRALIA

## Abstract

Strategic ejaculation is a behavioural strategy shown by many animals as a response to sperm competition and/or as a potential mechanism of cryptic male choice. Males invest more mating resources when the risk of sperm competition increases or they invest more in high quality females to maximize their reproductive output. We tested this hypothesis in the false garden mantid *Pseudomantis albofimbriata*, where females are capable of multiply mating and body condition is an indicator of potential reproductive fitness. We predicted male mantids would ejaculate strategically by allocating more sperm to high quality females. To determine if and how males alter their ejaculate in response to mate quality, we manipulated female food quantity so that females were either in good condition with many eggs (i.e. high quality) or poor condition with few eggs (i.e. low quality). Half of the females from each treatment were used in mating trials in which transferred sperm was counted before fertilisation occurred and the other half of females were used in mating trials where fertilisation occurred and ootheca mass and total eggs in the ootheca were recorded. Opposed to our predictions, the total number of sperm and the proportion of viable sperm transferred did not vary significantly between female treatments. Male reproductive success was entirely dependent on female quality/fecundity, rather than on the number of sperm transferred. These results suggest that female quality is not a major factor influencing postcopulatory male mating strategies in *P*. *albofimbriata*, and that sperm number has little effect on male reproductive success in a single mating scenario.

## Introduction

According to Darwin’s theory of sexual selection, females are the choosy sex whereas males compete with each other for female mates [1]. However, it was subsequently suggested that males might also become choosy when the associated costs of reproduction are high; for example when parental investment is significantly higher for males [2] or if females vary greatly in quality [3,4]. Intrasexual competition and male mate choice may continue during and/or after copulation in the form of sperm competition [5] or cryptic male choice [6,7]. These postcopulatory selective forces shape male mating strategies and are predicted to influence male reproductive success. Strategic ejaculation, where males tailor the ejaculate in response to variation in potential reproductive benefits [8], has been given much attention in the sperm competition and male mate choice literature during the last decade. Strategic ejaculation is likely to have evolved mainly as a defensive mechanism to overcome sperm competition [9–11], but also as a potential method of postcopulatory mate choice, where males cryptically invest more ejaculate into high quality females [12]. Although the sperm competition and cryptic male choice hypotheses are independent, it is important to note that they are not mutually exclusive. Indeed, except in rare instances where females are sperm limited, differential ejaculate expenditure as a result of cryptic male choice will coincide with sperm competition.

Sperm competition models predict that males should increase their mating investment when the risk of sperm competition (i.e. probability of competition) increases [11,13,14], but that they should decrease their mating investment when the intensity of sperm competition (i.e. absolute number of competitors) increases [9]. Male-male competition, female mating history and female quality have been identified as the most probable indicators of sperm competition and likely relate to the subsequent potential for strategic ejaculation [8,15]. Males of some species do indeed manipulate their ejaculate when competitors are present before or during copulation [16–20]. Risk and intensity of sperm competition can vary greatly with female mating history and the average mating rate of females in a particular mating system. Therefore, as suggested by Engqvist and Reinhold [10], males should invest comparatively more sperm in virgin females when the mating rate is high and when the mating rate is low they should invest more sperm in already mated females. And finally, female quality is associated with sperm competition because body condition may predict a female’s likelihood of re-mating with other males: higher quality females are more likely to receive attention from additional males than lower quality females. Empirical evidence coming mainly from insects indicates that males tailor their ejaculate strategically in response to female quality, presumably to counteract the risk or intensity of sperm competition [18,20–25].

Cryptic male choice plays a significant role in strategic ejaculation in animals [4,12]. It is more likely to evolve in response to the indicators of female fecundity, genetic compatibility, relatedness or female mating status (reviewed in [7]). Female quality is an indicator of potential reproductive ability/output of a particular female and can be measured in several ways such as age, size, condition or fecundity [4]. Since high quality females will have a higher potential reproductive fitness [6,26,27], it is selectively beneficial for males to invest more mating resources into those females that maximise reproductive success. For example, male scorpionfly *Panorpa cognata* produce larger salivary masses when copulating with highly fecund females, and females feed on the salivary masses during copulation [28]. Therefore, by increasing the size of the nuptial gift, males may copulate for longer and subsequently transfer more sperm.

Praying mantids are an ideal group in which to investigate the response to sperm competition and/or cryptic male choice because females are capable of multiple copulations [29–31], females store sperm in a sac-shaped spermatheca [32], and numerous males are often simultaneously attracted to the pheromones emitted by a single female [27,33–35]. However, very few studies have attempted to determine if and how male mantids respond to the risk of sperm competition (but see [36,37]). Prokop and Vaclav [36] found that male *Mantis religiosa* increase their copulation duration when the perceived risk of sperm competition is high (i.e. when the sex ratio is male biased). Copulation duration is often linked to the number of sperm transferred (reviewed in [6]), so the authors suggest that male *M*. *religiosa* mantids do indeed respond to the level of sperm competition risk. However, the link between copulation duration and sperm number is mostly relevant for species in which males transfer free-flowing sperm, so these results should be considered with caution. To date, there has only been one study that has directly tested the effect of sperm competition on strategic sperm allocation in praying mantids. Allen et al. [37] found that false garden mantids *Pseudomantis albofimbriata* transfer more sperm when the perceived risk of sperm competition is high (i.e. when the sex ratio is male biased), indicating that males are indeed capable of strategic ejaculation when they perceive an increased risk of intrasexual competition. Studies that investigate other factors (i.e. female mating status and female quality) are still required to fully understand the range of male responses in praying mantids.

In the current study, we used the sexually cannibalistic false garden mantid *Pseudomantis albofimbriata* to investigate the effect of female quality (i.e. body condition/fecundity) on male ejaculatory expenditure and reproductive success. Female quality is a major factor shaping the precopulatory mating strategies of this species [26,34,38–40]. Female body condition is positively related to female fecundity and negatively related to the propensity for cannibalism [26], and therefore males prefer good quality females in both close range (visual or chemical cues; [39]) and long distance (only chemical cues; [34,40]) mate choice trials. However, little is known about the influence of female quality on postcopulatory male mating strategies in this species. Therefore, our primary objective in this study was to determine whether male mantids allocate their sperm strategically in response to female quality. We predict that male *P*. *albofimbriata* will allocate more mating resources to high quality females via (1) the total number of sperm transferred, and/or (2) the proportion of viable sperm transferred. Our second objective was to link the amount of sperm transferred with actual reproductive success by comparing egg sac mass, total eggs in the egg sac and the percentage of eggs in the egg sac between high and low quality females. Finally, we determined whether male traits had an effect on sperm transfer or on actual reproductive success.

## Methods

### Study species and study sites

The false garden mantid *Pseudomantis albofimbriata* is a common mantid species distributed throughout Eastern Australia. Juvenile mantids (*N* = 130) were collected in December 2013-January 2014 from Ku-ring-gai Bicentennial Park, West Pymble (33^0^ 45' 37.76"S, 151^0^ 08' 20.88"E) and Yamba Reserve, Ryde (33^0^ 49' 0" S 151^0^ 6' 0"E), Australia. All individuals were found on the leaves and flowers of *Lomandra longifolia* bushes.

### Ethics statement

No permits were obtained for the described field collections/studies because New South Wales state law does not require specific permissions for the collection of invertebrates from locations outside of a national park. The study did not involve endangered or protected species.

### Rearing, sexing and measuring mantids

Mantids were reared individually within inverted transparent plastic cups of which the bottom end was replaced by mesh to facilitate better air flow. They were reared at a controlled temperature (25–26°C), light (14h) and humidity (55%) in laboratory conditions. Juvenile mantids were fed with two small crickets *Acheta domesticus* (body mass = 0.040 ± 0.001 g, *N* = 20) three times per week until maturity and watered daily. Once they became adults the pronotum length (i.e. fixed size) and mass were recorded, and individuals were sexed using differences in wing morphology. After adult eclosion, males continued with the usual diet of two small crickets three times per week, whereas females were allocated to one of the two feeding regimes: high quantity or low quantity. Females on the high quantity treatment (*N* = 30) were given three small crickets three times per week, and individuals on the low quantity treatment (*N* = 32) received one small cricket three times per week. Both males and females were weighed immediately preceding mating trials using digital scales. Body condition was calculated as body mass divided by fixed size, and was used as a measure of female quality.

### Mating trials

Mating trials were conducted in a large, high-ceilinged laboratory during the morning hours (0830–1130) in February-March 2014, and individuals were introduced onto a potted *Lomandra sp*. plant. First, the female was introduced onto a plant and was given five minutes to settle into the novel environment. Then a male was introduced 5 cm behind the female and precautions were taken to avoid cannibalism. Copulation was interrupted 3.5 hours after intromission (spermatophore transferred at ~ 3 hours [37]) and both the female and male were reweighed. After successful mating, approximately half of the females from each feeding treatment were used in sperm transfer trials (*N* = 29) and the other half were used in reproductive success trials (*N* = 33). In order to measure sperm number, females had to be euthanized and dissected which precluded collecting data on fertilisation rates. There was no significant difference in female body condition between sperm transfer trials (0.057 ± 0.004 g/mm) and reproductive success trials (0.053 ± 0.003 g/mm; Mann-Whitney *U* test: *Z* = -0.120, *N* = 62, *P* = 0.905).

### Aim 1. Do males allocate more sperm to high quality females?

The age of individuals was controlled so that on the day of the trial, females were 25.31 ± 0.65 (*N* = 29) days post-adult emergence and males were 26.17 ± 1.09 (*N* = 29) days post-adult emergence. After mating and sperm counting was complete, the female was dissected (see below for methods), and the number of large mature and small immature eggs in both ovaries was counted under a stereomicroscope. On the day of the mating trial, high food quantity treatment females were in significantly better condition (0.080 ± 0.003 g/mm, *N* = 14) than low food quantity females (0.035 ± 0.001 g/mm, *N* = 15, *t*-test: *t*_27_ = 12.95, *P* < 0.001). High quantity females had also produced significantly more mature eggs (91.29 ± 6.8) than low quantity females (22.33 ± 4.5; Mann-Whitney *U* test: *Z* = -4.596, *N* = 29, *P* < 0.001). Further, female body condition significantly predicted the number of mature eggs found in a female’s ovaries (linear regression: b = 1453.80, *r* ^2^ = 0.773, *F*_1, 28_ = 91.787, *P* < 0.001). Therefore, high quantity and low quantity female groups were renamed as ‘high quality’ and ‘low quality’ respectively.

After successfully mating, the female was immobilized with CO2 gas and dissected along the mid-dorsal line from posterior to anterior without damaging the interior parts. Her abdomen was fully opened by pinning the body wall to a wax tray. Next, the spermatophore was isolated and placed into a 1.5 mL Eppendorf tube containing 30 μL of Grace’s Insect Medium (Grace’s Insect Medium, Supplemented (×1), liquid, Life Technologies, Carlsbad, CA 92008). The spermatheca was then located and separated using fine forceps and placed onto a wax board containing 30 μL of Grace’s Insect Medium. The spermatheca was macerated very gently with small pins under the dissecting microscope. After maceration, the contents of the spermatheca was pipetted out with the Grace’s Insect Medium into a 1.5 mL Eppendorf tube. An additional 70 μL of Grace’s Insect Medium was added to the spermatheca to make the total dilution 100μL. The spermatheca was soaked for 10 minutes in Grace’s Insect Medium, allowing the majority of sperm to come into solution [41]. Twenty μL of the spermatheca sample was pipetted into a new 1.5 mL Eppendorf tube, taking care not to transfer large clumps into the new tube. The solution was then stained with 5 μL of diluted Propidium Iodide (PI) (11.25 μL of PI (1mg/mL) + 3.75 μL of Grace’s Insect Medium) for 15 mins in order to discriminate between live and dead sperm. Five μL of Synergy Brands (SYBR) Green 1 nucleic acid gel stain (1 μl SYBR Green 1 (10,000× concentrate in DMSO) + 49 μl of Grace’s Insect medium) was added to the same sample and left for 10 mins. After that, 10 μL of the sample was pipetted out into a haemocytometer (Neubauer improved double net ruling SVZ2NIOU) and live/dead sperm were counted in all grid squares under a fluorescent microscope. Live and dead sperm were identified easily as live sperm fluoresced in green and dead sperm fluoresced in red. The same procedure was repeated to count the number of sperm within the spermatophore, except it was macerated by three quick ruptures with fine forceps. The total number of sperm found in the spermatheca and the spermatophore were calculated separately by dividing the sum of live and dead sperm by grid volume (0.9μL) and then multiplying by the dilution factor (100μL). Finally, the total number of sperm transferred by each male was calculated by adding the number of sperm found in the spermatheca to the number of sperm in the spermatophore.

### Aim 2. Do males mating with high quality females gain higher reproductive success?

The age of individuals was controlled so that on the day of the experiment, females were 24.03 ± 0.55 (*N* = 33) days post-adult emergence and males were 21.94 ± 0.79 (*N* = 33) days post-adult emergence. Immediately preceding mating trials, high food quantity treatment females were in significantly better condition (0.070 ± 0.003 g/mm, *N* = 16) than low food quantity females (0.037 ± 0.001 g/mm, *N* = 17, Mann-Whitney *U* test: *Z* = -4.827, *N* = 33, *P* < 0.001). Since female body condition positively correlates with fecundity in praying mantids ([26,34,42] current study), high food quantity and low food quantity female groups were renamed as ‘high quality’ and ‘low quality’ respectively. After successful mating, the pair was labelled as mated individuals and placed back into their individual plastic cups. Females continued with their original feeding treatment and were allowed to lay an ootheca (egg sac). Once laid, the number of days taken to lay an ootheca and its mass were recorded. In addition to absolute ootheca mass, we used the percentage of eggs in the ootheca as an additional measure of male reproductive success. Therefore, once laid, females were dissected under a stereomicroscope and the number of large dark yellow coloured mature eggs (immature eggs can be easily distinguished as they are small and white or pale yellow in colour) remaining in her ovaries were counted. A few weeks later, oothecae were also dissected and the total number of eggs in each ootheca was counted under a stereomicroscope. Finally, the proportion of total eggs in the ootheca was calculated by dividing the total number of eggs in the ootheca by the total number of mature eggs produced by the female (i.e. total eggs in the ootheca plus the number of mature eggs remaining in her ovaries).

### Aim 3. Does male phenotype affect sperm transfer and/or male reproductive success?

We determined whether male phenotype (i.e. fixed size and condition) had an effect on sperm allocation (i.e. total number of sperm transferred and proportion of viable sperm) and to total eggs in the ootheca and the percentage of eggs in ootheca in *P*. *albofimbriata* by relating male traits to sperm allocation and to percentage of eggs in oothecae.

### Data analysis

Data were analysed using SPSS 21.0 for Windows and were checked for normal distribution (Kolmogorov-Smirnov test) before analysis. All non-normally distributed variables were log transformed and checked again for normality before proceeding. All stated values are mean ± standard error and all statistical tests are two tailed. General linear models (GLM) were used to determine the effect of female treatment and male phenotype on the total number of sperm transferred and the proportion of viable sperm (in sperm transfer trials). Effect sizes and confidence intervals (at the 0.05 level) for female quality and male phenotype were also generated. Regression analyses were performed to determine the effect of female body condition on the number of sperm transferred in sperm transfer trials. We performed a t-test to determine the difference in mean number of days taken by high quality and low quality females to lay their first ootheca in reproductive success trials. Mann-Whitney tests were carried out to compare the ootheca mass, total eggs in the ootheca and the percentage of eggs in the ootheca between female treatments in reproductive success trials. We performed non-parametric correlations to figure out the relationships among female body condition, weight of first oothecae and the total eggs in oothecae in reproductive success trials and also to relate male traits (i.e. fixed size and body condition) to total eggs in oothecae and percentage of eggs in oothecae.

## Results

### Aim 1. Do males allocate more sperm to high quality females?

**Fig 1:**
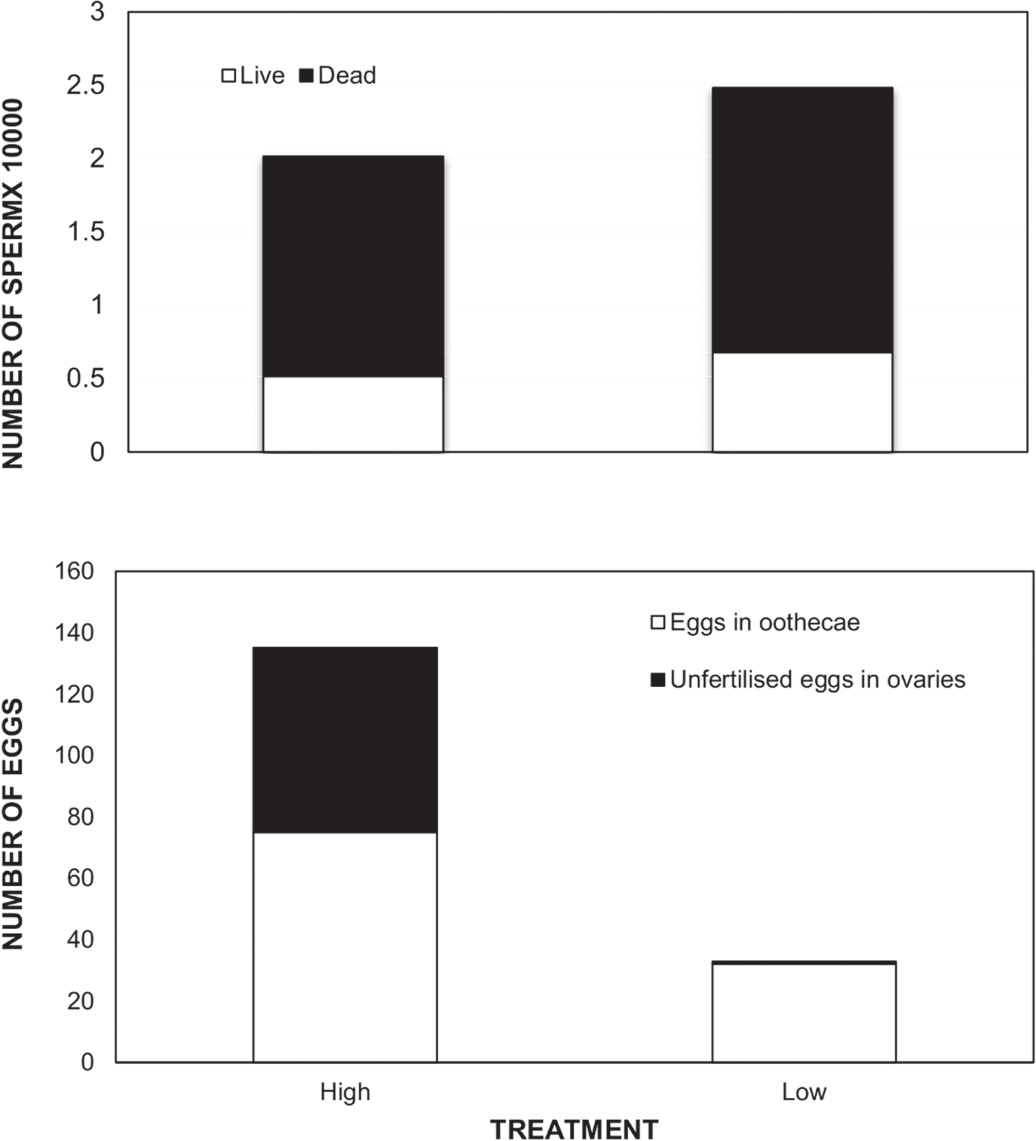
Comparison of Sperm transfer and proportion of eggs laid between female treatments. Female quality had no significant effect on the total number of sperm transferred by males (*P* = 0.301) or on the percentage of viable sperm (*P* = 0.958) in sperm trials. However, the percentage of eggs in oothecae was significantly different between female treatments (*P* < 0.001) in reproductive success trials, where low quality females laid a higher proportion of eggs than high quality females.

From the 29 trials, all males mounted females within the first hour of introduction and all males successfully transferred ejaculates to their mates within 3.5 hours. There was no significant difference in the log total number of sperm or the proportion of live sperm transferred (‘percent sperm viability’) between high quality and low quality females in *Pseudomantis albofimbriata* (Table 1; and see Fig 1). When comparing the variance (SE) as a percentage of the mean, variation of both the total number of sperm transferred (High quality -17.60%, Low quality—17.75%) and the percent sperm viability (High quality—14.69%, Low quality—14.33%) was very similar between treatments. We also found that effect sizes of female quality on log total number of sperm and on percent sperm viability were very close to zero and both confidence intervals overlapped zero, confirming that there was no effect of female quality on log total sperm transferred and on the percent sperm viability (Table 1). Female body condition did not predict the total number of sperm transferred across female treatments (linear regression: b = -2.035, *r* ^2^ = 0.036, *F*_1, 28_ = 0.996, *P* = 0.327) or within each female treatment (linear regression: [High quality] b = -0.575, *r* ^2^ = 0.001, *F*_1, 13_ = 0.007, *P* = 0.937; [Low quality] b = 14.429, *r* ^2^ = 0.089, *F*_1, 14_ = 1.273, *P* = 0.280).

**Table 1:** Effect of female quality on sperm allocation (total sperm number and percent sperm viability) and on reproductive success (days taken to lay an ootheca, ootheca mass and percentage of eggs in ootheca) in *P*.*albofimbriata*.

	High	Low	Statistics	Effect size	95% confidence intervals	
					Lower bound	Upper bound
**Log total sperm number**	4.21 ± 0.08	4.33 ± 0.06	*F*_1_ = 1.114, *P* = 0.301	0.043	-0.097	0.299
**Percent sperm viability**	26.24 ± 3.85	27.56 ± 3.95	*F*_1_ = 0.003, *P* = 0.958	0.001	-10.731	11.326
**Days to lay an ootheca**	4.50 ± 0.69	10.94 ± 1.31	*t*_*31*_ = -4.366, ***P* < 0.001**			
**Ootheca mass**	0.367 ± 0.03	0.135 ± 0.01	*Z* = -4.901**, *P* < 0.001**			
**Total eggs in ootheca**	75.00±5.31	32.06±1.98	*Z* = -4.867**, *P* < 0.001**			
**Percentage eggs in ootheca**	54.60 ± 1.85	96.41 ± 3.09	*Z* = -4.616, ***P* < 0.001**			

*F*-values were derived from general linear models using female treatment as a factor. Mann-Whitney tests were performed to compare the ootheca mass, total eggs in ootheca and percentage of total eggs in ootheca between female treatments. A *t*-test was used to compare the number of days taken to lay an ootheca between treatments. Significant *P* values are highlighted in bold.

### Aim 2. Do males mating with high quality females gain higher reproductive success?

All pairs (*N* = 33) were mated without sexual cannibalism and all females laid an ootheca successfully after mating. There was a significant difference in the number of days taken to lay the first ootheca between female treatments where high quality females laid ootheca significantly faster than low quality females (Table 1). Also high quality females produced a significantly heavier first ootheca compared to low quality females (Table 1). As expected, female body condition had a significant positive correlation with the mass of the first ootheca (Spearmen’s correlation: *r* ^2^ = 0.890, *N* = 33, *P* < 0.001). We also found a significant difference in total eggs within the ootheca between female treatments, where high quality females had significantly more eggs in the ootheca than low quality females (Table 1). Further, there was a significant positive correlation between female body condition and the total eggs in the ootheca (Spearmen’s correlation: *r* ^2^ = 0.877, *N* = 33, *P* < 0.001). The percentage of total eggs in the ootheca was significantly different between female treatments (Table 1). Furthermore, we found a significant positive correlation between ootheca mass and the total eggs in that ootheca (Spearmen’s correlation: *r* ^2^ = 0.958, *N* = 33, *P* < 0.001).

### Aim 3. Does male phenotype affect sperm transfer and/or male reproductive success?

There was no significant effect of male phenotype (fixed size and condition) on the total number of sperm transferred, or on the percentage of viable sperm transferred (Table 2). Further effect sizes of male phenotype on the total number of sperm and percent sperm viability were very close to zero and both confidence intervals overlapped zero, confirming the fact that there was no effect of male phenotype on either of the two variables (Table 2). We also found no significant effect of male phenotype on the total eggs in the ootheca and on the percentage of total eggs in ootheca (Table 2).

**Table 2:** Effect of male phenotype (fixed size and condition) on sperm allocation and reproductive success in *P*. *albofimbriata*.

	Male phenotype	Statistics	Effect size	95% confidence intervals	
				Lower bound	Upper bound
**Log total sperm number**	Fixed size	*F*_1_ = 0.116, *P* = 0.736	0.005	-0.144	0.202
	Condition	*F*_1_ = 2.749, *P* = 0.110	0.099	-89.003	9.509
**Percent sperm viability**	Fixed size	*F*_1_ = 0.073, *P* = 0.790	0.003	-10.841	8.397
	Condition	*F*_1_ = 1.931, *P* = 0.177	0.072	-4612.640	868.722
**Total eggs in oothecae**	Fixed size	P = 0.105	0.287		
	Condition	P = 0.884	-0.026		
**Percentage of eggs in oothecae**	Fixed size	*P* = 0.240	-0.210		
	Condition	*P* = 0.575	0.101		

*F*-values were derived from general linear models using male fixed size and condition as covariates. *P*-values for the relationships between male traits with total eggs in ootheca and the percentage of eggs in ootheca were derived from non-parametric correlations.

## Discussion

Contrary to prediction, we found no effect of female quality on the ejaculatory expenditure of male praying mantids, *Pseudomantis albofimbriata*. That is, males did not significantly vary the total number of sperm or the proportion of viable sperm transferred when paired with a high quality or a low quality female. Effect sizes and confidence intervals confirm these results. Variation in the total number of sperm and percentage of viable sperm was also very similar between treatments. Further, we found no significant relationship between female body condition and the total number of sperm transferred by males. Our finding is inconsistent with the general pattern shown for many insect groups in which males allocate their ejaculate strategically in response to female quality [18,20,24,25].

Since polyandry in *P*. *albofimbriata* has not been directly observed in the wild (but see [29]) and mated females become chemically unattractive to males soon after mating [29], it may be that polyandry, and hence sperm competition, is not a common occurrence and therefore males do not respond to its perceived risk. However, in *P*. *albofimbriata* [34] and in praying mantids more generally [33,35], multiple males may be simultaneously attracted to the initial pheromone plume of a single female, increasing the likelihood of multiple mating. Furthermore, a recent study found that males strategically ejaculate in response to the perceived risk of sperm competition associated with sex ratio biases [37], which suggests male *P*. *albofimbriata* do sense and respond to sperm competition via strategic ejaculation in certain contexts. Therefore, the absence of strategic ejaculation by male mantids in the current study is most likely due to female quality being a poor indicator of the risk of sperm competition in this system. Since praying mantids (including *P*. *albofimbriata*) exhibit a scramble competition polygynous mating system where males race to find virgin females and often arrive simultaneously [29,34,43,44], inter-male competition may act as a more appropriate indicator of the risk of sperm competition in this system. Alternatively, it may be that male mantids need longer than the 3.5 hours afforded to them in the current study to perceive sperm competition risk and modulate their ejaculate accordingly (see [18,20,45]). The treatments used in the aforementioned study showing strategic ejaculation in *P*. *albofimbriata* [44] were continued for an extended period during development (for nearly two weeks) and therefore, males had more time to modulate spermatophore production in response to the mating context.

Female condition is generally a good indicator of female fecundity in insects [4] and males may be able to achieve greater reproductive success by selectively allocating more sperm to good condition females as they produce more eggs that may require more sperm to fertilise them. Male mantids (including *P*. *albofimbriata*) have a preference for good-condition females rather than poor-condition females in a precopulatory scenario [27,34,39], but surprisingly males did not show a similar preference in the postcopulatory context during the current study. Differences in precopulatory and postcopulatory male mate choice have been observed in some other insect groups, for example male bush crickets *Kawanaphila nartee* prefer larger females in precopulatory choice [46], but transfer more sperm to smaller females [24]. In the current study (and see [34,47]), high quality females produced a significantly higher number of mature eggs compared to low quality females, and males transferring the least number of sperm (total sperm 3444) still transferred many more than was necessary to fertilize the most fecund female’s egg load (total eggs 173). Therefore, it may not be selectively beneficial for males to allocate more sperm to high quality females, but still important to find a high quality female in the precopulatory arena as she will be more fecund and less likely to cannibalise [26,48].

In our reproductive success trials, high quality females laid significantly heavier first oothecae within a shorter time period compared to low quality females. Poor condition females had fewer mature eggs in their ovaries compared to good condition females ([34] current study), hence, it is likely that poor quality females delay laying the ootheca to allow more time for further egg production. As expected we found a significant positive correlation between female body condition and first ootheca mass—this result is consistent with previous studies conducted on the same species [26] and on the praying mantid *Hierodula membranacea* [42]. Further, we found significant positive correlations between female body condition and total eggs in the ootheca and also between ootheca mass and total eggs in the ootheca. Also high quality females put significantly more eggs in their ootheca than low quality females. These results confirm that female condition has a significant effect on the reproductive output of praying mantids [26,42]. The percentage of eggs in the ootheca was significantly different between female treatments. That is, low quality females laid almost all available mature eggs and high quality females laid only half of their mature eggs (the other half of mature eggs remained unfertilised in their ovaries). Poor condition females may fertilise more mature eggs because they are less attractive to males [27,39] and are therefore less likely to gain additional mating opportunities. High quality females may instead prefer to partially delay fertilisation so as to obtain additional mates, thereby increasing the genetic diversity of offspring [31]. Alternatively, high quality females might simply choose to lay multiple oothecae so as to literally not put ‘all of their eggs in one basket’, thereby reducing the risk of offspring predation. We found no significant link between the total number of sperm transferred and any of the indicators of actual reproductive success (i.e. first ootheca mass and number of eggs in ootheca), demonstrating a disconnect between sperm transfer and reproductive success for males in a single mating scenario. Reproductive success for male *P*. *albofimbriata* is mostly dependent on female condition/fecundity in a single-male scenario, however sperm number is likely to have an effect on reproductive success if females mate multiply. According to the fair raffle principle, males who transfer more sperm should gain a higher proportion of fertilisations in a multiple mating scenario ([49,50] reviewed in [11]).

Sperm allocation may vary with male phenotype [51–53]. For example in domestic crickets *Acheta domesticus*, sperm number correlates with male body size/pronotum length [52] or body weight [51]. However, we found no evidence for a relationship between male phenotype and sperm allocation in *P*. *albofimbriata* (see [54]). Effect sizes and 95% confidence intervals confirmed these results. We also found no significant effect of male phenotype on total eggs in the ootheca and on percentage of eggs in ootheca in reproductive success trials. Therefore it is unlikely that females bias fertilisation, favouring males with specific phenotypes. However, some studies have shown that females may exhibit cryptic choice based on male phenotype and favour the sperm of males with specific traits [55,56]. The lack of an effect of male phenotype on sperm transfer and also on fertilisation success in the current study may be due to minimal variation in male size and body condition, as we did not manipulate male body size/condition. Future studies that manipulate male size and condition will better determine if such a relationships exists in *P*. *albofimbriata*.

In summary, the current study suggests that male *P*. *albofimbriata* mantids do not strategically adjust their ejaculate in response to female quality, providing evidence of a disconnect between their precopulatory and postcopulatory strategies. Furthermore, the amount of sperm transferred does not play a significant role in the reproductive success of males in a single mating context. Male phenotype had no effect on sperm allocation or on reproductive success in *P*. *albofimbriata*. Future studies focusing on strategic ejaculation in a multiple mating scenario will be important in gaining a more complete understanding of postcopulatory male mating strategies in praying mantids.

## Supporting Information

S1 Table Underlying (raw) sperm transfer trials data. (CSV)

S2 Table Underlying (raw) reproductive success trials data. (CSV)
