# Correction: The Effect of Female Quality on Male Ejaculatory Expenditure and Reproductive Success in a Praying Mantid

**DOI:** 10.1371/journal.pone.0137814

**Published:** 2015-09-02

**Authors:** Anuradhi Jayaweera, Katherine L. Barry

The Funding section is incorrect. The complete, correct Funding statement is: The Joyce W. Vickery Scientific Research Fund from the Linnean Society of NSW (http://linneansocietynsw.org.au/grants.html) funded this research. The funder had no role in study design, data collection and analysis, decision to publish, or preparation of the manuscript.
